# Evidence for the evolutionary origin of goldfish derived from the distant crossing of red crucian carp × common carp

**DOI:** 10.1186/1471-2156-15-33

**Published:** 2014-03-15

**Authors:** Jing Wang, Shaojun Liu, Jun Xiao, Min Tao, Chun Zhang, Kaikun Luo, Yun Liu

**Affiliations:** 1 Key Laboratory of Protein Chemistry and Fish Developmental Biology of Education Ministry of China, College of Life Sciences, Hunan Normal University, Changsha 410081, China

**Keywords:** Goldfish, Red crucian carp, Common carp, Distant hybridization, Evolution

## Abstract

**Background:**

Distant hybridization can generate transgressive hybrid phenotypes that lead to the formation of new populations or species with increased genetic variation. In this study, we produced an experimental hybrid goldfish (EG) by distant crossing of red crucian carp (*Carassius auratus*) × common carp (*Cyprinus carpio*) followed by gynogenesis.

**Results:**

We evaluated the phenotype, ploidy level, gonadal structure, and 5S rDNA of the EG. Diploid EG possessed a high level of genetic variation, which was stably inherited. In particular, the EG combined transgressive phenotypes, including a forked tail and shortened caudal peduncle, traits that are present in common goldfish. The EG and common goldfish share a number of morphological and genetic similarities.

**Conclusions:**

Using the EG, we provide new evidence that goldfish originated from hybridization of red crucian carp × common carp.

## Background

**Figure 1:**
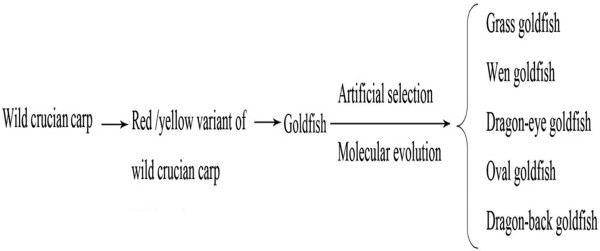
Hypothesis for the formation of goldfish.

Goldfish (*Carassius auratus* var.) belong to the order Cypriniformes and are regarded as a variant form of crucian carp (*Carassius auratus*). However, the evolutionary origin of goldfish is obscure. Previous studies have hypothesized that goldfish originated from a wild population of crucian carp. It was proposed that red/yellow individuals were derived from wild crucian carp through natural mutation. This variant is thought to have split into several lines, including the grass goldfish (long tail), the wen goldfish (forked tail), dragon-eye goldfish (bulging eye), oval goldfish (no dorsal fin), and dragon-back goldfish (bulging eye and no dorsal fin) [1,2] (Figure 1). Goldfish exhibit a large range of phenotypic diversity. They are generally distinguished from crucian carp by their body color and shape, and the appearance of the back, tail, anal fin, head, eyes, scales, opercula, and nares film. One of the more recognizable features of the goldfish is the bifurcated tail. In contrast, crucian carp have an undivided tail. Currently, there are more than 240 goldfish lines cultured in the world. Despite the interest in the evolutionary history of goldfish, there is a lack of direct evidence to support the hypothesis that they are derived from wild crucian carp. Interestingly though, recent studies of mitochondrial gene sequences provide support for this hypothesis [3-5]. In this study, we developed a new goldfish variant using distant hybridization to provide further insight into the evolutionary origin of goldfish.

**Figure 2:**
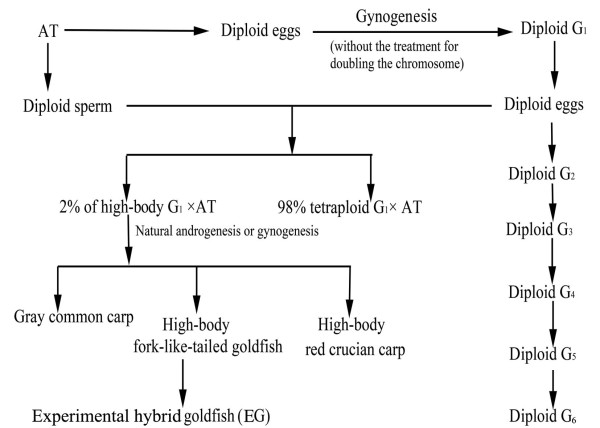
**Formation of EG.** AT: Allotetraploid derived from distant hybridization of red crucian carp × common carp, G_1–6_: Gynogenesis progeny of the allotetraploid.

An allotetraploid (AT) hybrid was first obtained by crossing red crucian carp (RCC; *C. auratus* red var., ♀, 2n = 100) with common carp (CC; *Cyprinus carpio* L., ♂, 2n = 100) [6,7]. AT individuals produce diploid eggs and diploid sperm. Gynogenesis and androgenesis have been applied to the diploid gametes produced by AT. Using these techniques, a gynogenetic diploid hybrid clone line (G_1_–G_2_–G_3_–G_4_–G_5_–G_6_) [8-10] and an androgenetic diploid hybrid clone line were established [11,12]. The gynogenetic diploid hybrids produce diploid eggs, a unique reproductive trait. Fertilizing the diploid eggs produced by G_1_ with diploid sperm from male ATs yielded a new tetraploid hybrid (G_1_ × AT, 4n = 200). This new species has a number of advantages over AT individuals, including greater disease resistance, more desirable phenotype (e.g., higher body, smaller head, and shorter tail), higher fecundity, and faster growth rate [13]. Importantly, the G_1_ × AT population consists of two groups. The first, consisting of 98% of G_1_ × AT progeny, are tetraploids. The appearance of these fish is similar to that of the AT. The remaining 2% of the G_1_ × AT progeny have a ratio of body height to body length of 0.48 (versus ≈ 0.36 for the majority). Self-mating of the high-body G_1_ × AT produces three forms of bisexual fertile diploid fish: high-body red crucian carp, high-body fork-tailed goldfish, and gray common carp [14]. We established a population of experimental hybrid goldfish (EG) (F_1_–F_2_–F_3_–F_4_–F_5_–F_6_) by the self-mating of the fork-tailed goldfish (Figure 2). Goldfish that experienced long-term natural variation and artificial selection are referred to as naturally domesticated goldfish (NG).

We determined the phenotype, ploidy level, and gonadal structure of EG individuals. In addition, we evaluated the relationship between EG, NG, RCC, and CC using 5S rDNA. Our results provide insight into the evolutionary origin and diversification of goldfish, and the role of distant hybridization during transgression and speciation.

## Results

### Formation of the EG population

The process of EG formation is illustrated in Figure 2. The self-crossing of EG was associated with high fertilization rates (average 90.60%) and hatching rates (average 86.52%). The progeny of EG maintained the parental traits, including the double tail and variable coloration and markings. The body color of the progeny began to differentiate in 1-month-old goldfish and stabilized at age three months.

### Comparison of morphological traits among EG, NG, RCC, and CC

**Figure 3:**
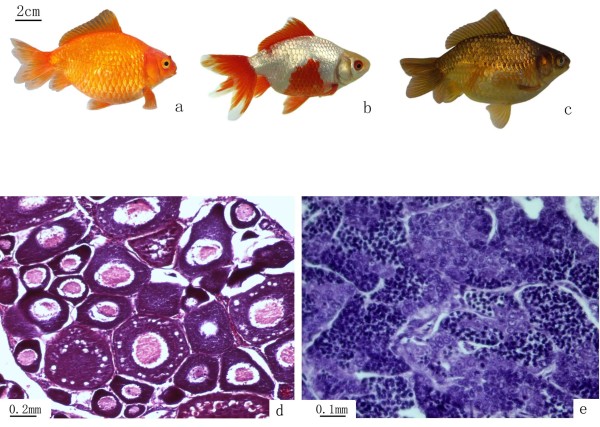
**Illustration of the variants of EG individuals and gonadal structure of EG. (a)** Red individual; **(b)** mixed color individual; **(c)** black individual; **(d)** III-stage ovary of EG, containing II-phase and III-phase oocytes; **(e)** mature testis of EG, containing mature spermatozoa, spermatids, and spermatogonia.

Compared with RCC and CC, EG possessed several unique phenotypes, including a forked tail, spherical body, short caudal peduncle, and a range of body coloration (e.g., red, black, white, and mixed colors) (Figure 3a, b, c).

**Figure 4:**
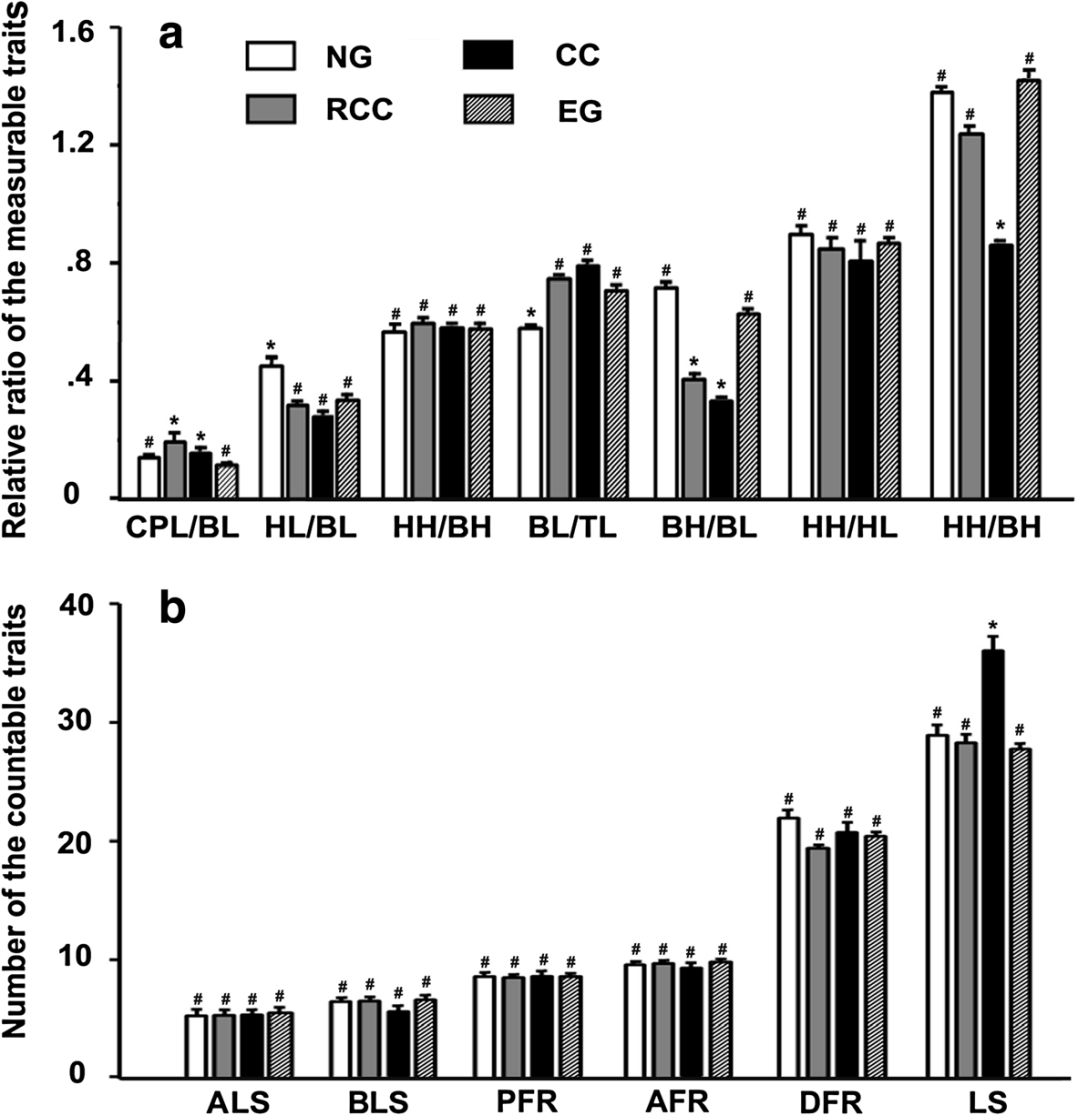
**Measurable and countable traits of EG, NG, RCC, and CC. (a)** Ratios of the measurable traits. **(b)** Counts of countable traits. For each trait, mean values that are labeled with different symbols (# or *) are significantly different (*P* < 0.05). TL, total length; BL, body length; BH, body height; HL, head length; HH, head height; CPL, caudal peduncle length; CPH, caudal peduncle height; LS, lateral line scales; ALS, scale rows above the lateral line; BLS, scale rows below the lateral line; DFR, dorsal fin rays; PFR, pelvic fin rays; AFR, anal fin rays.

There was no significant difference (*P* > 0.05) between NG and EG individuals for all traits, except for the ratios of head length to body length and body length to total length (Figure 4a). However, ratios of body height to body length and caudal peduncle length to body length differed significantly (*P* < 0.05) between EG and RCC and between EG and CC. Thus, the morphological characteristics of EG were very similar to those of NG, but differed from those of its original parents RCC and CC. For example, the ratios of body height to body length were 0.63 and 0.72 for EG and NG individuals, respectively, whereas RCC and CC individuals had much smaller ratios (0.41 and 0.34, respectively; Figure 4b). We found no significant differences in countable traits among EG, NG, and RCC, although the lateral line scale count in CC (36.0) was significantly different from that in the other three variants (EG 27.7, NG 28.9, RCC 28.2).

### Chromosome number and DNA content in EG individuals

**Figure 5:**
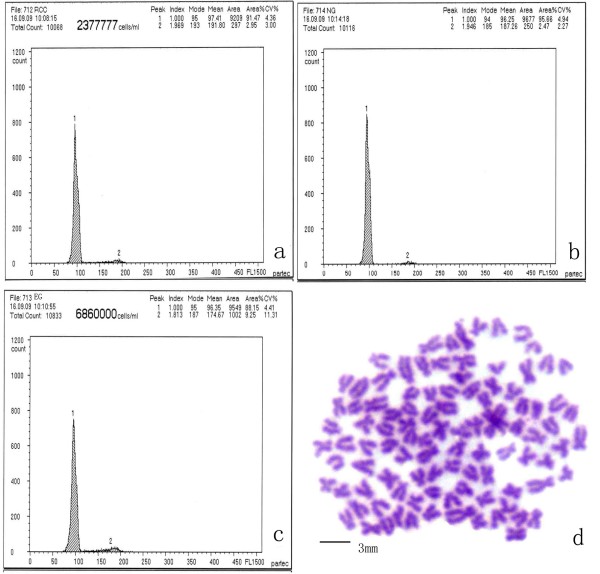
**Chromosome number and DNA content of EG individuals.** DNA content of RCC **(a)**, EG **(b)**, and NG **(c)**; **(d)** metaphase chromosome spread of EG (2n = 100).

EG individuals had the same DNA content as the control groups (diploid RCC and NG) (Figure 5a, b, c; no significant difference (*P* > 0.05) between EG (mean: 96.35) and NG (96.25), or between EG and RCC (97.41). The chromosome number in 100 metaphase spreads ranged between 95 to 100 for the majority of EG individuals (93%), suggesting that most EG are diploid (2n = 100) (Figure 5d).

### Gonadal microstructure of EG

One-year-old EG individuals were able to produce normal mature gametes. We stripped white sperm from 7-month-old males and mature ova from 9-month-old females. Observation of the gonadal tissue sections revealed that the ovaries of 5-month-old EG females were at the III-stage, indicating that EG are fertile (Figure 3d). In the testes, we observed numerous mature spermatozoa, spermatids, and spermatogonia in the seminiferous tubules (Figure 3e).

### Analysis of 5S rDNA PCR

**Figure 6:**
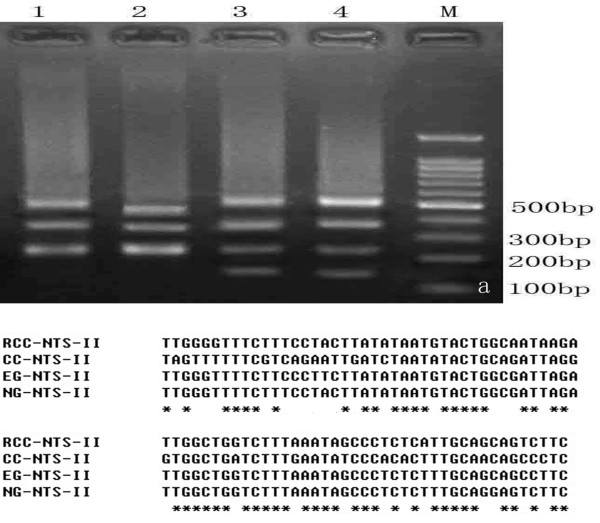
**DNA band patterns and phylogenetic trees. (a)** DNA band patterns of 5S rDNA in RCC, CC, NG and EG. 1, RCC yielded three DNA fragments (approximately 200, 340, and 500 bp); 2, CC yielded three DNA fragments (approximately 200, 300, and 400 bp); 3, NG yielded four DNA fragments (approximately 150, 200, 340, and 500 bp); 4, EG yielded four DNA fragments (approximately 150, 200, 340, and 500 bp); M, DNA ladder markers with an interval of 100 bp. **(b)** Comparison of NTS-II sequences from RCC, CC, EG and NG. Asterisks indicate the consensus bases.

Using the 5S primer pair, DNA fragments were amplified from RCC, CC, EG, and NG. These fragments generated distinct agarose gel electrophoresis band patterns. There were three PCR fragments (approximately 200, 340, and 500 bp) in RCC, three PCR fragments (approximately 200, 300, and 400 bp) in CC, and four PCR fragments (approximately 150, 200, 340, and 500 bp) in EG and NG (Figure 6a). To further evaluate differences in 5S rDNA patterns, a total of 140 clones were sequenced, including 30 clones from RCC, 30 clones from CC, 40 clones from EG, and 40 clones from NG (10 clones for each PCR fragment). We obtained three different sizes (203, 340, and 477 bp) in RCC, three in CC (203, 317, and 414 bp), four in EG (168, 203, 340, and 492 bp), and four in NG (168, 203, 354, and 501 bp). Based on BLASTn analysis, all fragments from RCC, CC, EG, and NG were confirmed as 5S rDNA repeat units. All the sequences were submitted to GenBank (see Table 1 for accession numbers).

**Table 1:** GenBank accession numbers of the 5S rDNA sequences in EG, NG, RCC and CC

	**GenBank accession no. of the sequences**			
	**EG**	**NG**	**RCC**	**CC**
168 bp	GU127593	GU188688	–	–
203 bp	GU127594	GU186887	GU205788	GU188691
≈340 bp	GU127595	GU188689	GU205789	GU188692
≈500 bp	GU127596	GU188690	GU205790	GU188693

The 5S rDNA unit is a moderately repetitive sequence with a highly conserved coding region (120 bp) and a nontranscribed spacer (NTS) region. The sequences of the 5 s rDNA units cloned in this study contained a coding region (5′-99 bp and 3′-21 bp) and a mid-region consisting of distinct NTS sequences. In EG, the four monomeric 5S rDNA classes (designated class I: 168 bp; class II: 203 bp; class III: 340 bp, and class IV: 492 bp) were characterized by distinct NTS types (designated NTS-I, NTS-II, NTS-III, and NTS-IV for the 48, 83, 220, and 372 bp sequences, respectively). In NG, the four monomeric 5S rDNA classes (designated class I: 168 bp; class II: 203 bp; class III: 354 bp, and class IV: 501 bp) were characterized by distinct NTS types (designated NTS-I, NTS-II, NTS-III, and NTS-IV for the 48, 83, 234, and 381 bp sequences, respectively). In RCC, the three monomeric 5S rDNA classes (designated class II: 203 bp; class III: 340 bp; and class IV: 477 bp) were characterized by distinct NTS types (designated NTS-II, NTS-III, and NTS-IV for the 83, 220, and 357 bp sequences, respectively). In CC, the 414 bp sequence was a dimer of the 203 bp sequence. So in CC, only two monomeric 5S rDNA classes (designated class II: 203 bp and class V: 317 bp) were characterized by distinct NTS types (designated NTS-II: 83 bp and NTS-V: 197 bp). The NTS region was characterized by polymorphism of its length and high genetic variability. Therefore, we were able to analyze the genetic relationship between the groups by comparing the similarity of their NTS regions. Pairwise sequence comparisons among RCC, CC, EG, and NG (Table 2) revealed that EG shared high homology with NG. Both EG and NG had a unique NTS-I, which was not detected in RCC or CC. The sequence comparison of NTS-II among EG, NG, RCC, and CC suggested that EG and NG contained RCC and CC specific bases (Figure 6b). A comparative analysis of NTS-III and NTS-IV sequences suggested there were several base substitutions or insertions–deletions among RCC, EG, and NG (poly A sequences were inserted to EG-NTS-IV, NG-NTS-III, and NG-NTS-IV) However, the CC characteristic class NTS-V was not observed in EG or NG.

**Table 2:** Nucleotide similarities of NTS sequence among CC, RCC, NG, and EG

**NTS types**	**EG/RCC**	**EG/CC**	**EG/NG**	**NG/RCC**	**NG/CC**
NTS-I	Absent in RCC	Absent in CC	97.9%	Absent in RCC	Absent in CC
NTS-II	91.5%	72.2%	95.1%	93.9%	70.2%
NTS-III	95.9%	Absent in CC	82.5%	82.9%	Absent in CC
NTS-IV	88.4%	Absent in CC	94.5%	86.4%	Absent in CC

## Discussion

### Significance of distant crossing and gynogenesis in genetic breeding

Distant crossing introduces a heterogeneous complete genome into the ovum, resulting in the recombination of distantly related genes. This, in turn, may cause changes in the regulation and expression of genes in the hybrid progeny [15-18]. The process of EG formation involves the crossing of two parents, RCC and CC, which belong to different genera. When the haploid egg of the RCC is fertilized with haploid sperm from the CC, the fusion of the male and female nuclei forces the integration of the two genomes resulting in the formation of diploid offspring (F_1_–F_2_) and, following later crossings, a tetraploid offspring (F_3_–F_20_). These hybrid offspring have notable differences in appearance from the parent species. For example, RCC have no barbel whereas the CC have two pairs of long barbels. The hybrid progeny of these two species have two pairs of short barbels. Furthermore, many of the morphometric characteristics of the hybrid progeny are intermediate between RCC and CC (e.g., body color, scale type, number of pharyngeal teeth). Diploid eggs and spermatozoa produced by AT are heterozygous gametes. Gynogenesis using diploid eggs does not require treatment to double the number of chromosomes to produce a generation of gynogenetic fish. These offspring possess hybrid traits in appearance, and the unique reproductive trait of producing diploid eggs. The diploid eggs produced by G_1_ were fertilized with diploid sperm from AT to produce a new tetraploid (G_1_ × AT). It was of interest that 2% of the G_1_ × AT population exhibited the high-body trait. This character differentiation was not observed in 20 generations (F_3_–F_22_) of AT hybrids. Self-mating of the high-body G_1_ × AT progeny produced three diploid variants: a high-body RCC, a high-body fork-tailed goldfish, and a gray common carp. All three variants were bisexual and fertile. However, it is unclear how these variants were derived. We speculate that natural gynogenesis or androgenesis occurred during the mating process between G_1_ × AT. In addition, natural androgenesis may have played an important role in the formation of bisexual fertile individuals. Furthermore, the formation of these three variants illustrates that the combination of distant crossing and gynogenesis is likely to produce diverse progeny. The formation of high-body fork-tailed goldfish suggests that there is a close genetic relationship between goldfish and RCC or CC.

Artificial distant hybridization and gynogenesis are analogous to the accidental phenomena of natural gynogenesis and hybridization. Because crucian carp and common carp occupy the same habitat, accidental mating may occur resulting in some viable hybrid offspring. Although distant hybrids are difficult to propagate by self-mating, natural gynogenesis may produce offspring (e.g., triploid silver Prussian carp).

### Formation and analysis of morphological traits

The forked tail was a unique trait of EG, which differed from both parents but was also characteristic of NG. Goldfish may also be distinguished from crucian carp by the shortened caudal peduncle. The ratio of caudal peduncle length to body length was 0.12 in EG, which is similar to that for NG (0.14) but lower than that for RCC (0.20) or CC (0.16). The hybrids frequently expressed trait values exceeding the range between the parental means, which is referred to as transgressive segregation [19,20]. It is thought that transgression in hybrids is often caused by complementary gene action or epistasis [20-22]. In our study, the EG possessed a bifurcated tail, shortened caudal peduncle, and markings and coloration similar to those of NG, all features that differed from its parents. These transgressive phenotypes were readily inherited in the progeny of EG. However, other morphological features characteristic of NG (e.g., the dorsal fin, operculum, and eye) were not differentiated in the EG progeny. There were a number of variations of the tail fin, body color, and shape in EG individuals, but no differentiation of the dorsal fin, anal fin, and eye. If our model of hybrid origin of goldfish is correct, we surmise that during the evolutionary history of goldfish, the tail fin and body color may have differentiated before other characteristics such as the fin shape, head type, eye type, number of scales, and the opercula. Komiyama [5] hypothesized that the process of artificial selection began with the loss of the dorsal fin followed by diversification of other characteristics, such as the shape of eyes. Currently, the formation of goldfish variants is the result of both molecular evolution and artificial selection.

### Evidence of molecular biology

A number of researchers have studied the origin of goldfish. Based on investigations of embryonic development [23], chromosome karyotypes [24], lactate dehydrogenase and esterase isozyme amplification patterns [25,26], muscle protein electrophoresis bands [27], serum antigen reactions [28], random amplified polymorphic DNA markers [29], and mitochondrial DNA [30], it is believed that goldfish and crucian carp share a close genetic relationship and are the same species. Despite these studies, there are differing views regarding the evolutionary origin and history of goldfish [5,31,32]. Komiyama constructed a phylogenetic tree based on eight gene regions in the mtDNA of goldfish, crucian carp, and common carp. Based on the tree, the authors concluded that goldfish in China and Japan all originated from the Chinese crucian carp [5].

In this paper, we used 5S rDNA to study the genetic relationships among various fish. The molecular evolution of 5S rDNA is linked to the vertical inheritance of species. During the process of hybridization, and concomitantly with recombination of the genome, new classes of 5S rDNA units are produced by derivation from parental 5S rDNA. Given this, 5S rDNA have been widely used to infer the relationships among different species using comparative sequence data. We used 5S rDNA primers to amplify the genomic DNA from NG, EG, and the original parents. Our results demonstrated that EG shared the same expansion bands as NG, including four monomeric 5S rDNA classes (class I: 168 bp; class II: 203 bp; class III: ≈340 bp, and class IV: ≈500 bp). The class I sequence differed between RCC and CC. We speculate that the novel fragment was formed by gene recombination during the hybridization of RCC × CC. This was because the distant crossing of RCC and CC first generated diploid progeny, then tetraploids, followed by gynogenesis. Last, through long-term genetic selection, the variant returned to the diploid form. During this process there was likely to have been a high degree of gene recombination and chromosome exchange. Thus, EG possess a new DNA sequence not present in the original parents. The class II was a heterozygous sequence that contained bases specific to the two original parents. However, the characteristic paternal class IV was not observed in EG. Several studies have reported parental genome-specific loss of ribosomal DNA following allopolyploid formation [33-37]. For example, the paternal 5S rDNA sequences are absent in the genome of hybrid red crucian carp × blunt snout bream [38]. It is hypothesized that the fertility of hybrids is improved by reducing the amount of incompatible parental genetic material by genetic recombination, mutations, and removal of DNA. Such a process would explain the appearance of new 5S rDNA sequences and the loss of paternal 5S rDNA in the genome of hybrids [37,38]. The process of EG formation includes an allopolyploid generation which may explain the loss of the CC class V sequence in EG. The high similarity of 5S rDNA sequences in EG and NG indicate that, besides being similar in appearance, EG and NG are also highly similar at the molecular level. Based on these observations, we hypothesized that NG and EG share a common origin.

## Conclusions

Researchers have proposed a number of hypotheses explaining the evolutionary relationship among goldfish, crucian carp, and common carp based on phenotypic traits and physiological and biochemical data. We used distant hybridization and gynogenesis to produce an EG. The formation of EG provided a unique opportunity to study the origin and evolution of goldfish.

In summary, tetraploid hybrids may be generated by crossing of distantly related species, female RCC and male CC. These tetraploids can produce many kinds of diploid fish by gynogenesis or androgenesis. These diploids are similar to their primitive parents but not identical, which indicates that changes in ploidy by genetic breeding can yield a variety of offspring with differing ploidy levels. Moreover, distant crossing can generate transgression in hybrids.

## Methods

All samples, including gynogenetic diploid hybrids (G_1_), AT, improved tetraploid hybrids (G_1_ × AT), EG, RCC, and CC were cultured in ponds at the Protection Station of Polyploidy Fish, Hunan Normal University, and fed with artificial feed. NG were purchased at a local market (we chose wen goldfish with red body color and forked tails as the control group). Fish treatments were carried out according to the regulations for protected wildlife and the Administration of Affairs Concerning Animal Experimentation, approved by the Science and Technology Bureau of China. Approval from the Department of Wildlife Administration was not required for the experiments conducted in this paper. Fish were deeply anesthetized with 100 mg/L MS-222 (Sigma-Aldrich, St Louis, MO, USA) before dissection.

### Animals and Crosses

The improved tetraploids G_1_ × AT were formed by crossing diploid eggs of 2-year-old G_1_ with the diploid sperm of 1-year-old AT. The high-body individuals (2% of the progeny) from this cross were selected and self-crossed, producing a high-body fork-tailed goldfish. The EG population was then obtained by self-crossing of high-body fork-tailed goldfish. During the reproductive season (from April to June), mature paternal fish of EG were selected for self-mating. Mature eggs were fertilized with milt and the developing embryos were cultured in dishes at a water temperature of 19–20°C. About 3,000 EG embryos were taken at random to measure the fertilization rate (number of embryos at the gastrula stage/number of eggs × 100%) and hatching rate (number of hatched fry/number of eggs × 100%), five parallel experiments for each group. The hatched fry were transferred to a pond for further culture.

### Measurement of morphological traits

We randomly selected 40 1-year-old fish from each group (EG, NG, RCC, and CC) for morphological examination. We measured total length (TL), body length (BL), body height (BH), head length (HL), head height (HH), caudal peduncle length (CPL), and caudal peduncle height (CPH) in each fish (accurate to 0.1 cm). These values were then used to calculate the ratios: BH/BL, BL/TL, HL/BL, CPL/BL, HH/HL, CPH/CPL, and HH/BH. In addition, we recorded counts of the following variables: number of lateral line scales, number of scale rows above and below the lateral line, and the number of dorsal, anal, and pelvic fin rays. We used analysis of variance (ANOVA) and multiple comparisons (LSD-method) to test for differences in each trait among the four kinds of fish using SPSS Statistics 17.0 (IBM Corp. New York, USA). Basic graphical presentations were prepared using Sigma Plot 10.0 (San Jose, CA, USA). Values of independent variables are expressed as means ± SD.

### Examination of the ploidy level

We measured the DNA content of 30 1-year-old EG individuals. The diploid RCC and NG were used as control groups (5 individuals in each sample). We collected 0.5–1 ml blood from the caudal vein of each individual using a syringe containing ≈ 200–400 U sodium heparin. The blood samples were then filtered and stained with Cystain DNA 1 Step Staining Solution (Partec, Görlitz, Germany). The DNA content of each sample was measured by flow cytometry (Partec).

We prepared chromosome spreads using kidney cells that were extracted from 10 EG individuals. The fish were reared for 1–3 d at a water temperature of 18–22°C. Prior to dissection the fish were injected with concanavalin A (10 μg/g body weight). Each fish was given two injections at a 12 h interval. Each fish was also injected with colchicine (4 μg/g body weight) 3 h prior to dissection. The kidney tissue was ground in 0.9% NaCl and centrifuged for 1 min at 289 g. The cells were immersed in a hypotonic solution consisting of 0.075 M KCl for 40 min at 37°C and then fixed using 3:1 methanol/acetic acid (three changes). The cells were transferred to a cold and wet slide using a pipette, stained for 30 min in 4% Giemsa in phosphate buffer (pH 7.0), and observed under a light microscope using an oil lens. We photographed 10 metaphase spreads from each sample to determine the chromosome number.

### Observation of gonadal structure

To observe the gonadal structure, we selected 10 5-month-old EG. The gonads were fixed in Bouin’s solution for 24 h, dehydrated using an ethanol gradient, and cleared in xylene. The sections were embedded in paraffin, cut at 6–8 μm, and stained with hematoxylin and eosin. The microstructure was observed and photographed using a Pixera Pro 600ES (Pixera Corporation, Santa Clara, CA, USA). We identified the gonad stages based on standards for cyprinid fish [39].

### 5S rDNA PCR amplification, cloning, and sequencing

Total genomic DNA was isolated from the blood cells using a DNA extraction kit following the manufacturer’s instructions (Sangon, Shanghai, China). We synthesized the following primers: 5S rDNA F: 5′-GCTATGCCCGATCTCGTCTGA-3′ and 5′-CAGGTT GGTATGGCCGTAAGC-3′ to amplify the 5S rDNA from genomic DNA extracted from EG, NG, RCC, and CC. The amplification reaction mixture (25 μl) consisted of 20 ng genomic DNA, 1.5 mM MgCl_2_, 0.2 mM of each dNTP, 0.4 μM of each primer, 1× PCR buffer, and 1.25 U Taq polymerase (Takara, Dalian, China). The temperature profile during amplification was: initial denaturation at 94°C for 4 min, followed by 30 cycles of 94°C for 30 s, 60°C for 30 s, and 72°C for 1 min. A final extension step was performed at 72°C for 10 min. The PCR products were separated on a 1.2% agarose gel, purified using a Gel Extraction Kit (Sangon), ligated into a pMD18-T vector, and transferred into *E. coli* DH5a. The positive clones were then sequenced using an automated DNA sequencer (ABI PRISM 3730: Applied Biosystems, Carlsbad, CA). The sequences amplified from RCC, CC, EG, and NG were analyzed using ClustalW2 and Jellyfish software and submitted to GenBank.

## Competing interests

The authors declare that they have no competing interests.

## Authors’ contributions

The experiments were designed by SJL, YL and JW. Experimental organisms were cultured by JW and KKL. Studies of cellular characteristics were conducted by JX, MT and JW. Molecular biological investigations were carried out by JW and CZ. The manuscript was prepared by JW, JX and SJL with input from the other co-authors. All authors read and approved the final manuscript.
